# Distalization in Orthodontics: A Review and Case Series

**DOI:** 10.1155/2021/8843959

**Published:** 2021-01-20

**Authors:** Yahya A. Alogaibi, Ahmad A. Al-Fraidi, Manar K. Alhajrasi, Saleh S. Alkhathami, Abdulkarim Hatrom, Ahmed R. Afify

**Affiliations:** ^1^Bisha Dental Center, Ministry of Health, P.O. Box 418, Bisha 61922, Saudi Arabia; ^2^Department of Orthodontic, King Fahad Hospital, Specialized Dental Center, Madina, Saudi Arabia; ^3^Department of Orthodontic, North Jeddah Specialty Dental Center, MOH, Jeddah, Saudi Arabia; ^4^Department of Orthodontic, Ministry of Health, P.O. Box 4137, Makkah, Saudi Arabia; ^5^Orthodontic Department, Faculty of Dentistry, King Abdulaziz University, P.O. Box 80209, Jeddah 21589, Saudi Arabia; ^6^Orthodontic Department, Faculty of Dentistry, Mansoura University, Mansoura, Egypt

## Abstract

Distalization is a conservative method that is utilized in orthodontics to gain space by moving posterior teeth distally. It may be combined with other space gaining strategies, such as expansion, or can be used alone. Many methods have been used for distalization. These methods differ significantly in their place, whether to be extraoral or intraoral, site of action in upper and/or lower arch, and cooperation needed by the patient if it is removable or fixed. This review illustrates some of the most commonly used methods for distalization with a brief presentation of three cases that incorporated successful distalization techniques.

## 1. Introduction

Molar distalization is a term that is commonly used now for referring to the procedure of increasing the length of the dental arch by the rearward movement of the buccal segment teeth [1, 2]. For more than 100 years, maxillary molar distalization has been successfully used in orthodontics to treat many cases, most notably are cases with class II malocclusion. This technique is usually utilized to gain space to relieve crowding and to reduce the increased overjet [3]. A primary advantage in this technique is the ability to gain space in a conservative way without the need for extraction [4–6].

Other treatment options to gain space are expansion (which is commonly used in combination with distalization), proclination of anterior teeth, interproximal stripping, extraction, and orthognathic surgery. All these methods are available for orthodontic treatment but not all of them are suitable for every case. The severity of malocclusion and the facial profile usually govern these choices [7].

Class II malocclusion is one of the most frequent problems in orthodontics, and distalization is considered one of the conservative ways to treat such cases [1, 8]. Nonextraction treatment of class II malocclusion has become very popular in the last decade [9].

One of the common methods to achieve distalization is the incorporation of extraoral appliances such as headgears. These appliances are commonly utilized either for anchorage to support maxillary molars or for distalization of molars to correct the molar relation and to increase the arch length. These methods were found to be both efficient and reliable when used correctly [10, 11].

However, these appliances depend mainly on patient compliance and have a problem of bad esthetics, which led to the preference of intraoral distalizing appliances by some patients. Intraoral appliances are more accepted by the patient and allow more control for the orthodontist on teeth [12].

## 2. Search Strategy

A search was done on PubMed to find all the available literature on distalization and orthodontics. This search showed total articles of 556, which showed only 95 articles were related to this topic. The Endnote software (version X7) was utilized to file the articles and to remove duplicates.

### 2.1. Indications for Molar Distalization

- To mesially position the maxillary first molar
- Preferred in patients with low mandibular plane angle (brachy-cephalic type) or normal (meso-cephalic type)
- Mild to moderate class II molar relationship, which are not indicated for extraction
- To correct the second molar position
- To achieve ideal overbite and overjet
- To early correct class II pattern
- To regain the lost space (space regainer)

### 2.2. Contraindications for Molar Distalization

- Patients with high mandibular plane angle and excessive lower anterior facial height
- Patients with skeletal or dental open bite
- Severe class II skeletal pattern with an orthognathic maxilla and retrognathic mandible
- Excessive overjet and proclination of anterior teeth
- Crowding in the posterior segment
- Patients with temporomandibular joint problems

### 2.3. Various Modalities to Distalize Molars

#### 2.3.1. Extraoral Appliances

In 1892, Norman Kingsley described for the first time a device called headgear, which can achieve maxillary molar distalization. This was the beginning of the universal use of headgears [13]. The force utilized for molar distalization by headgears should be constant and steady to allow for effective teeth movements and should be relatively light as it is mainly concentrated on the first molars. The amount of the recommended force is about 100 grams, which allows for a rate of tooth movement of 1 mm per month. This duration of wear should be as long as possible as the more the patient will wear the appliance, the better and faster the expected results. However, the minimum recommended time for headgear is 14 to 16 hours daily [14, 15]. Orthopedic force, on the other hand, which is a force aiming to modify craniofacial bones, should be much higher than the orthodontic force required for tooth movement. These forces should be ranging from 150 to 300 gams [16].

Headgear type is usually determined according to the type of vertical problem. In cases of deep bite, cervical headgear is usually recommended to extrude the molars during distalization and thus correct the deep bite anteriorly. On the other hand, a case with open bite a high pull headgear is usually indicated as it leads to both intrusion and distalization of molars. Straight headgear can be utilized in cases where no vertical problems exist [17–19].

#### 2.3.2. Intraoral Appliances

There are many examples of intraoral distalizing appliances including but not limited to the following: Pend-X appliance, Lip Bumper, Forsus appliance, Herbst Appliance, Twin block, modified Nance holding arch, Japanese NiTi coils, molar distalizing system using magnets, Distal Jet, Pendulum appliance, Ghoshgarian TPA, Carriere appliance with class II elastics, and Sliding Jig with miniscrews combined with closed coil. However, not all of these appliances are commonly used among orthodontists. Only some of them were found to be more preferred to be used [3, 20].

*(1) Distalizing the Upper Arch (Class II Correctors)*. In 1992, an appliance called pendulum was described by Hilgers, which was found to be an efficient method for distalization the upper molars. This appliance consisted of palatal button similar to Nance holding arch, which is retained by wires bonded to the occlusal surface of premolars and two coil springs distal to the palatal button that are attached to the palatal sheaths of molar bands. These coils act as the active component for distalizing the molars. Soon after the introduction of this appliance, it was updated by adding an expansion screw inside the palatal button, which was named “Pend-X.” The Pend-X appliance was found to be a reliable method for distalization as it prevents the tendency of crossbite in molars during distalization by slight expansion of the arch. Also, it can be utilized in cases that need both arch expansion and distalization [21].

Forsus class II appliance is a newer version of functional appliance that has the ability for upper molar distalization. It is composed of a specially designed telescopic spring, which does not bend on the cheek, and its spring is comfortable and easily brushed and cleaned [22, 23].

Herbst appliance is a fixed functional device designed by Emil Herbst in 1909, and Pancharz popularized it in 1979. It is composed of bilateral telescopic device that can protrude the mandible causing a reactive force on maxillary molars leading to its distalization. This device can produce both skeletal and dental changes in growing patients [24].

Twin blocks are those appliances having upper and lower bite blocks that incorporate inclined planes on the occlusal surfaces. These inclined planes with a 70-degree angle exert a distal force on the upper posterior teeth that may lead to distalization. Twin block is well tolerated by patients and relatively smaller than many other functional appliances. It also has an advantage of having minimal interference with speech [25, 26].

*(2) Distalizing the Lower Arch*. Lip bumper is another functional distalizing appliance that is composed of a stainless steel wire covered labially by acrylic to avoid ulceration of the lower lip. This appliance is inserted in the tubes of the lower molars and extends anteriorly to contact the lower lip. The labial portion is positioned 2-5 mm from the lower incisors causing distal forces from the lower lip to the molars. It has also an effect on incisor proclination as it alters the equilibrium between the lower lip and the tongue [27, 28].

Active lingual arch is another effective method for molar distalization in the lower arch. It has many modifications that enable the operator to distalize the molars unilaterally or bilaterally. However, this may come on the expense of the lower anteriors as it may lead to a reactive incisor proclination. So this appliance is usually recommended when lower incisors are retroclined and molars are in need of distalization [29, 30].

Franzulum is another appliance, which was introduced by Buyoff et al., in the year 2000. It was composed of a button made of acrylic that is situated inferior and lingual to the lower canine on each side. Additionally, there are two rests situated on the first premolar and the canine. The appliance utilizes a nickel titanium coil spring as an active component to produce the forces of distalization [31].

*(3) Universal Distalizing Methods for Upper and/or Lower Arches*. Miniscrews have been reliably used as a method to gain absolute anchorage by either direct or indirect methods. Direct method of anchorage means that the active component causing the movement is directly attached to the miniscrew(s) while indirect attachment means that a tooth or teeth are attached to the miniscrew(s), and then, this tooth or teeth are utilized for anchorage. However, indirect anchorage was reported to be less reliable than direct anchorage as it may show some degree of anchorage loss. Nevertheless, miniscrews in general were able to efficiently distalize both molars and premolars without affecting the position of anterior teeth and minimal distal tipping [32, 33].

#### 2.3.3. First Case

**Figure 1:**
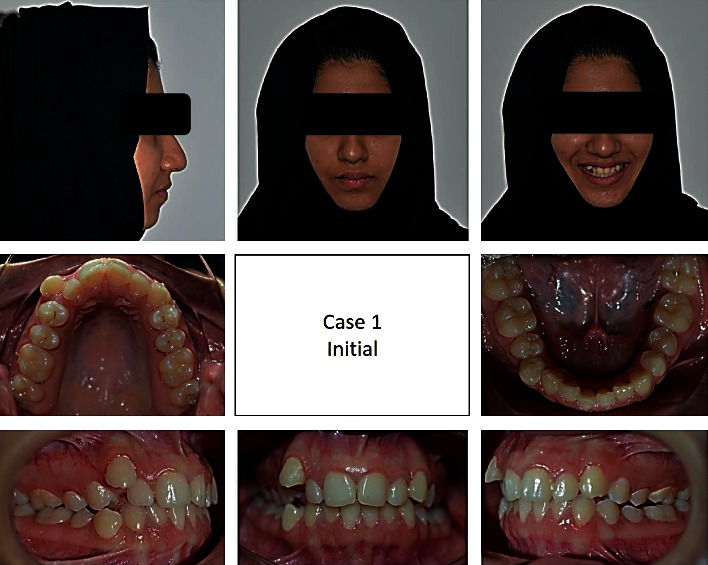
Pretreatment extraoral and intraoral photographs of the patient.

**Figure 2:**
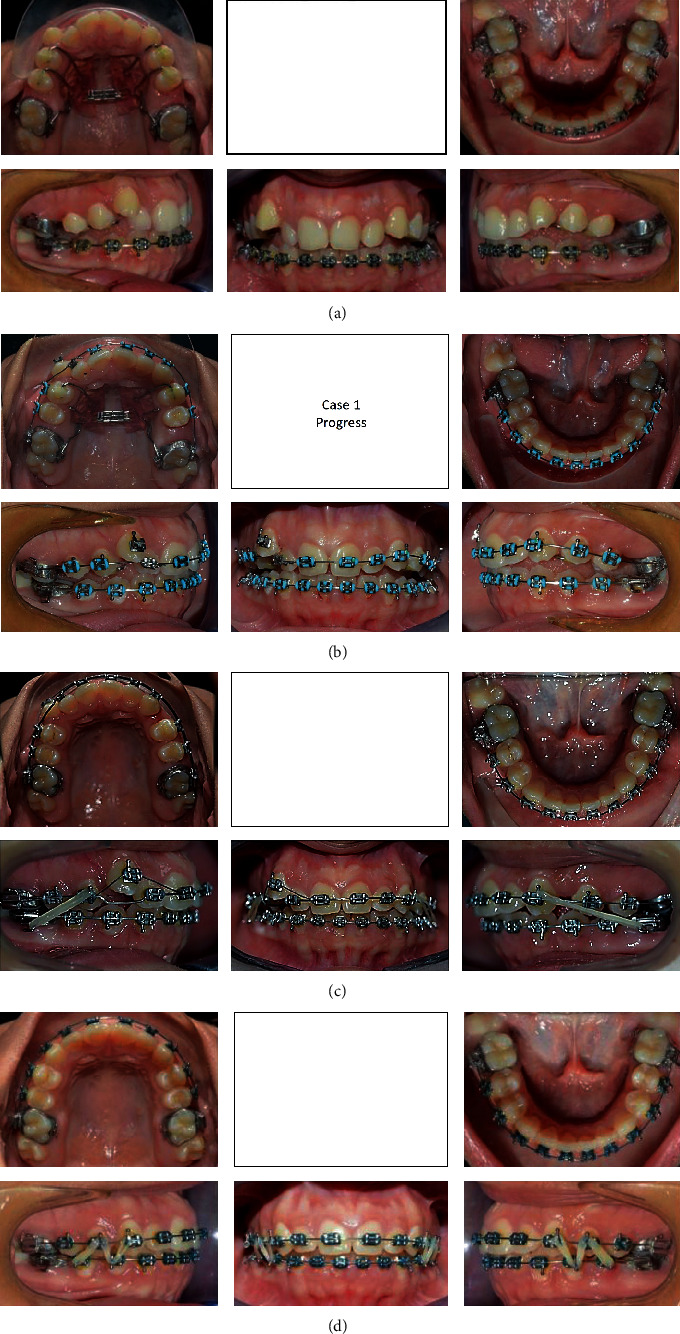
(a) Progress treatment intraoral photographs of the patient. (b) Progress treatment intraoral photographs of the patient. (c) Progress treatment intraoral photographs of the patient. (d) Progress treatment intraoral photographs of the patient.

**Figure 3:**
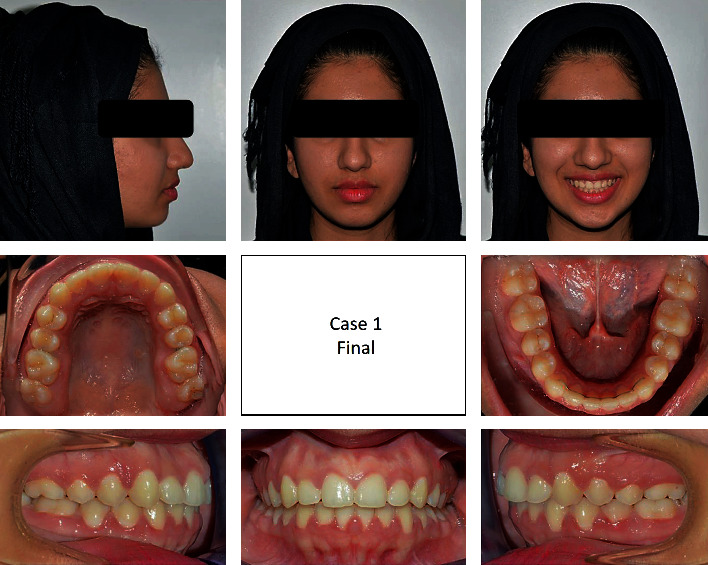
Posttreatment extraoral and intraoral photographs of the patient.

**Figure 4:**
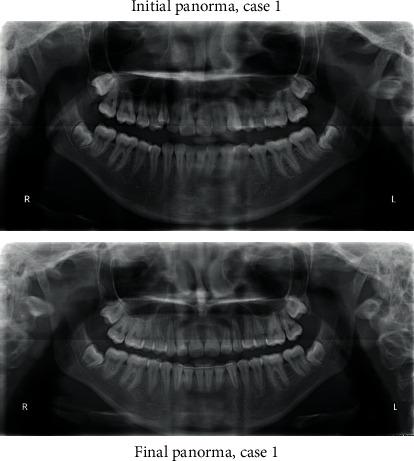
Initial and final panoramic radiographs.

**Figure 5:**
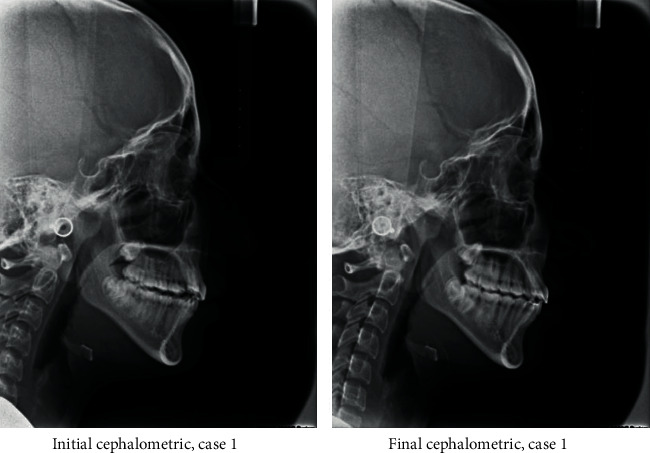
Initial and final cephalometric radiographs.

**Figure 6:**
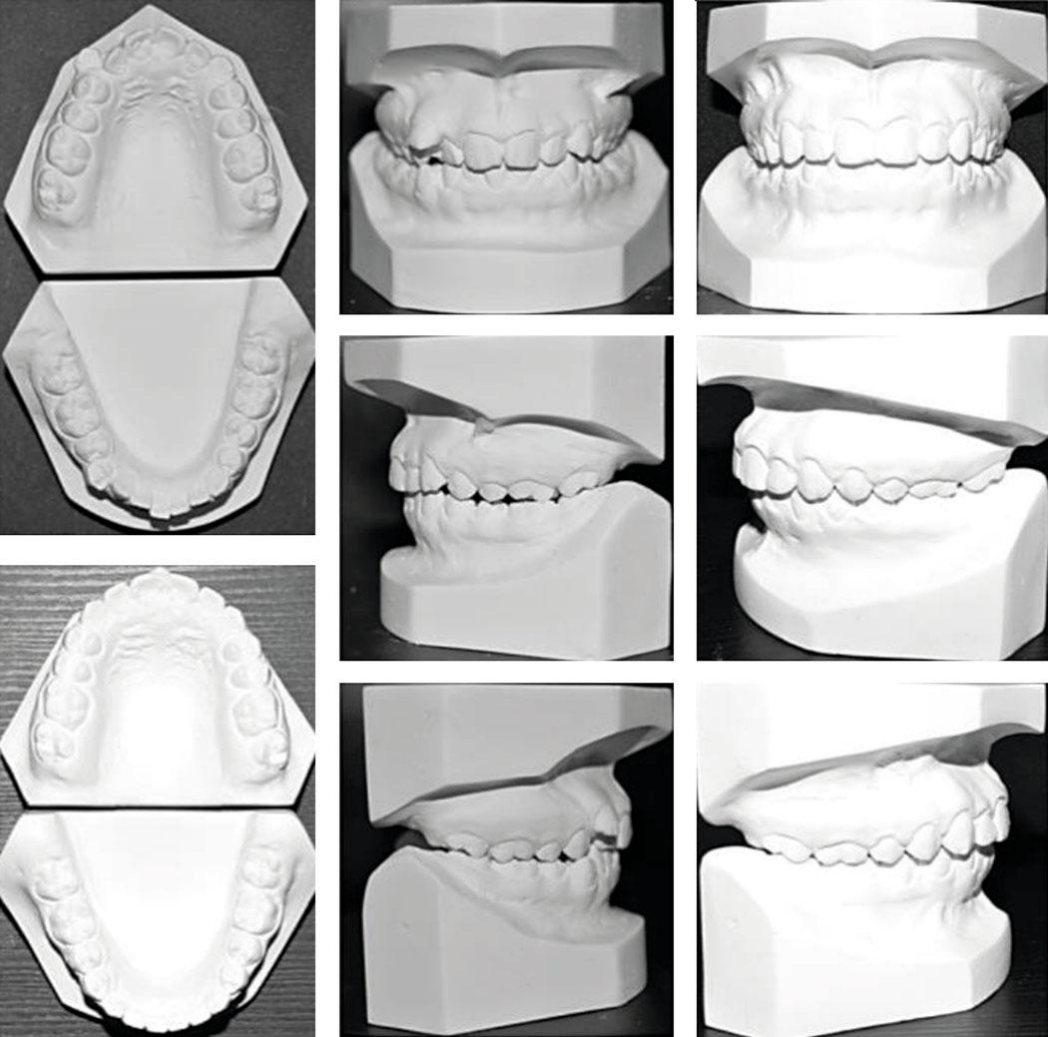
Initial and final study cast.

**Figure 7:**
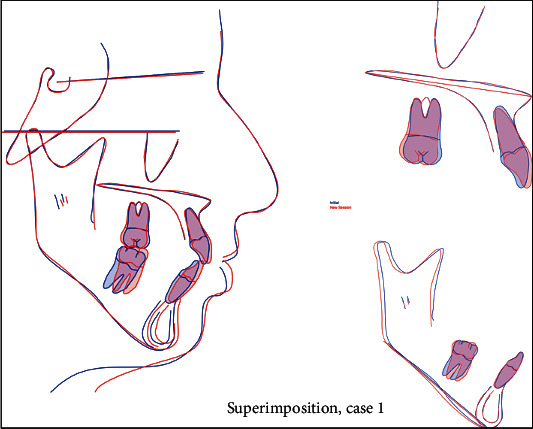
Superimposition.

A 17-year-old female presented with class I incisors relationship on class II skeletal base with average vertical proportions. Her chief complaint was from crooked teeth and high canine. The molar relationship was 1/2 unit class II at left side and 1/3 unit class II at right side. The upper midline was shifted to the right by 2 mm. The crowding in the upper arch was moderate and in the lower was mild. Treatment initially was held using Pend-X appliance for distalization. This was followed by fixed appliance (0.018^″^ slot size) Roth prescription, on nonextraction basis. Pend-X appliance was able to correct the molar relationship into class I relation and to gain a space to relief the incisor crowding and the ectopically erupted right canine by both expansion and distalization (Figures 1–7).

#### 2.3.4. Second Case

**Figure 8:**
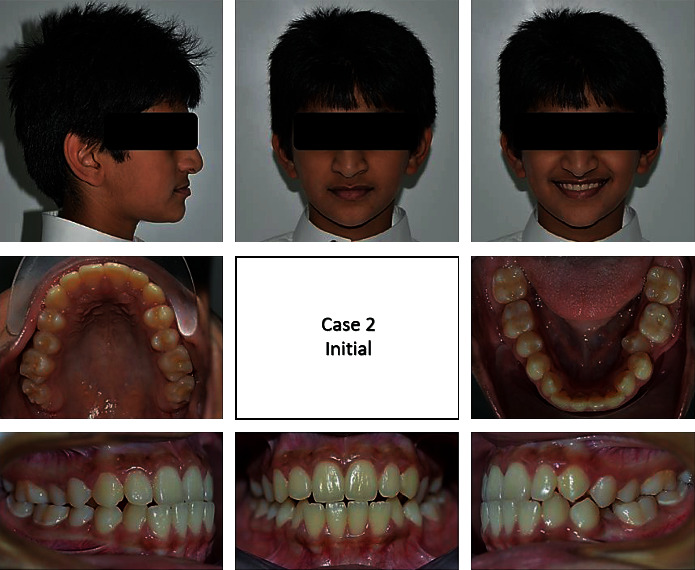
Pretreatment extraoral and intraoral photographs of the patient.

**Figure 9:**
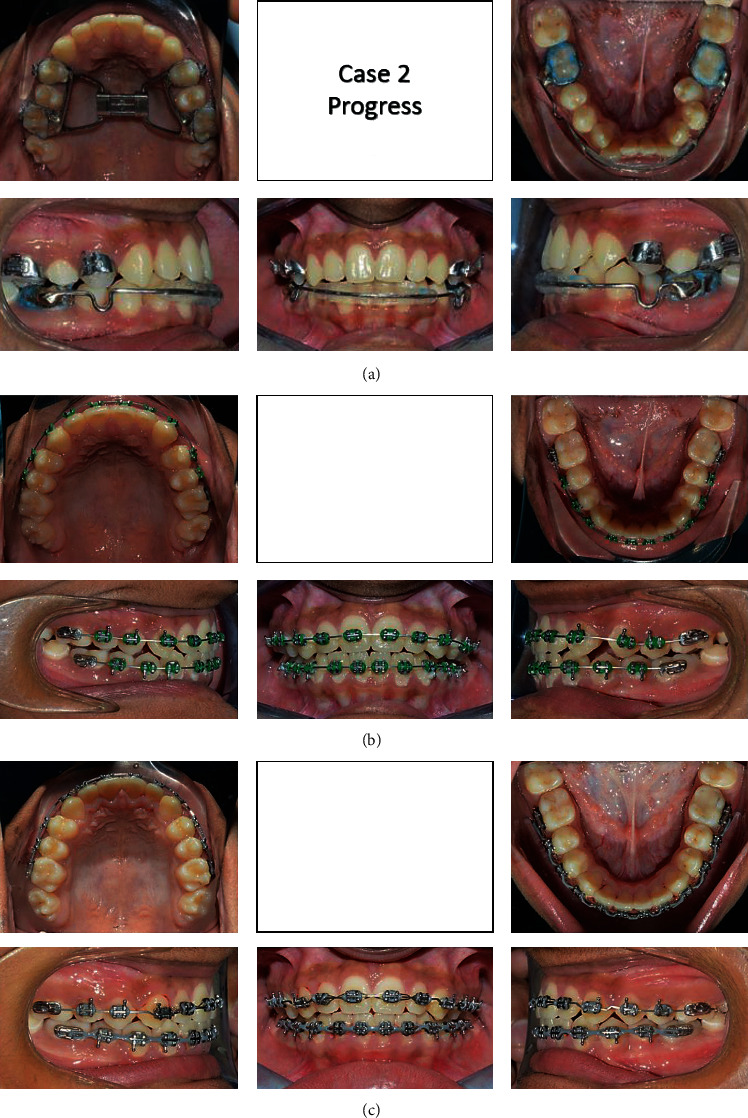
(a–c) Progress treatment intraoral photographs of the patient.

**Figure 10:**
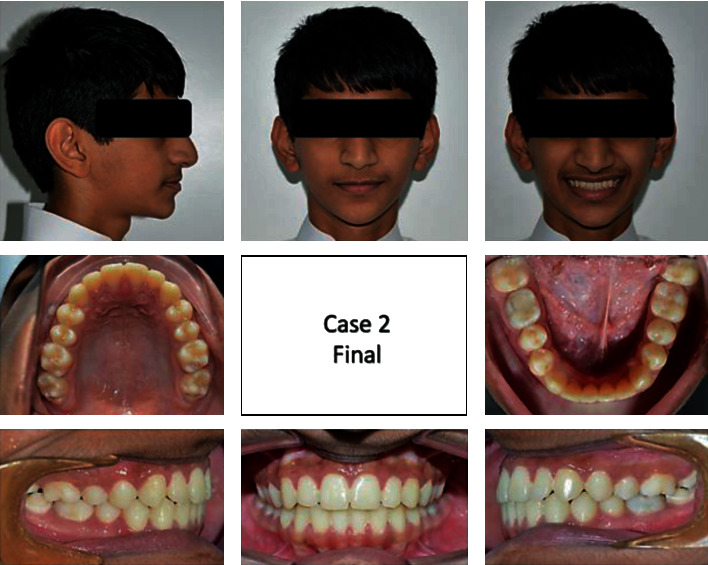
Posttreatment extraoral and intraoral photographs of the patient.

**Figure 11:**
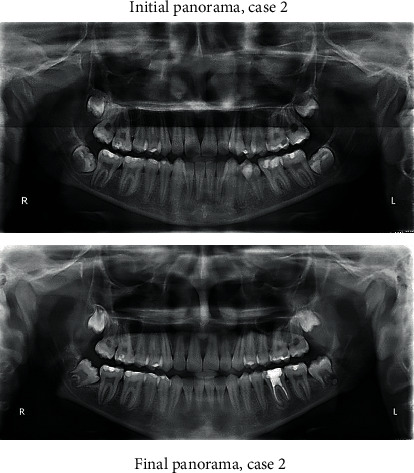
Initial and final panoramic radiographs.

**Figure 12:**
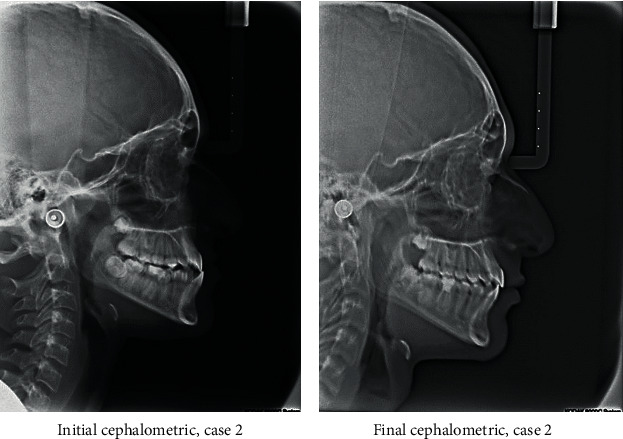
Initial and final cephalometric radiographs.

**Figure 13:**
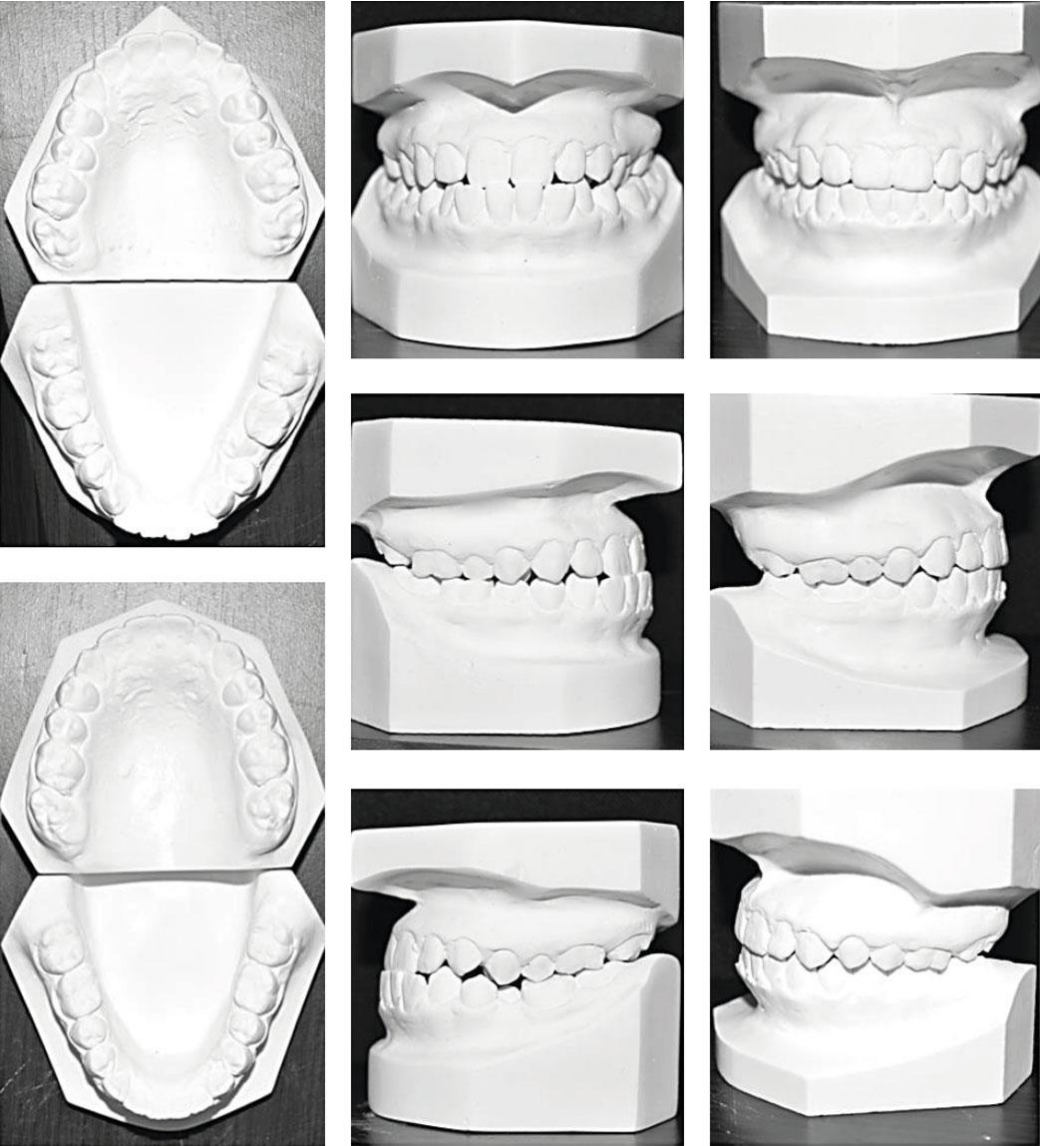
Initial and final study cast.

**Figure 14:**
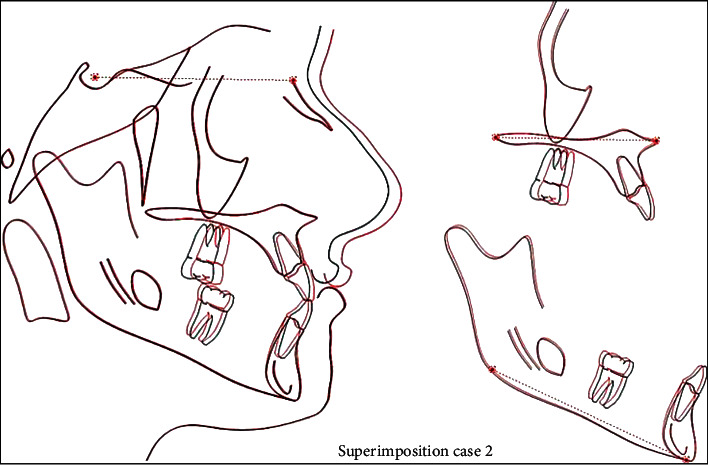
Superimposition.

A 13-year-old male came to the clinic with a chief complaint of unpleasant teeth. Upon examination, the patient revealed class III incisor relation with anterior crossbite, minimal overbite, and skeletal class III base with horizontal growth pattern. The patient had class III molar relation on both sides. The canine relation was class III on the right side and class I on the left side. The lower left second premolar was ectopically erupted to the lingual side. Treatment plane was aimed at protracting the maxilla forward and at distalizing the lower molars to correct the class III molar relation and to give a space for alignment of the lower left second premolar. Maxillary protraction was achieved by utilizing facemask combined with rapid maxillary expansion using hyrax to disarticulate the maxilla from its sutures. Lip bumper was utilized to distalize the lower molars and was able to successfully correct the class III molar relation and to gain a space for the lower left second premolar (Figures 8–14).

#### 2.3.5. Third Case

**Figure 15:**
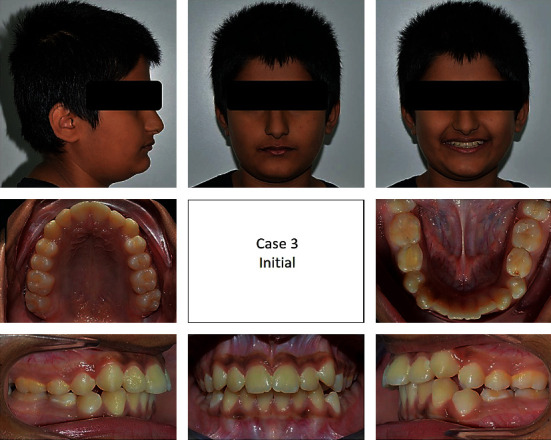
Pretreatment extraoral and intraoral photographs of the patient.

**Figure 16:**
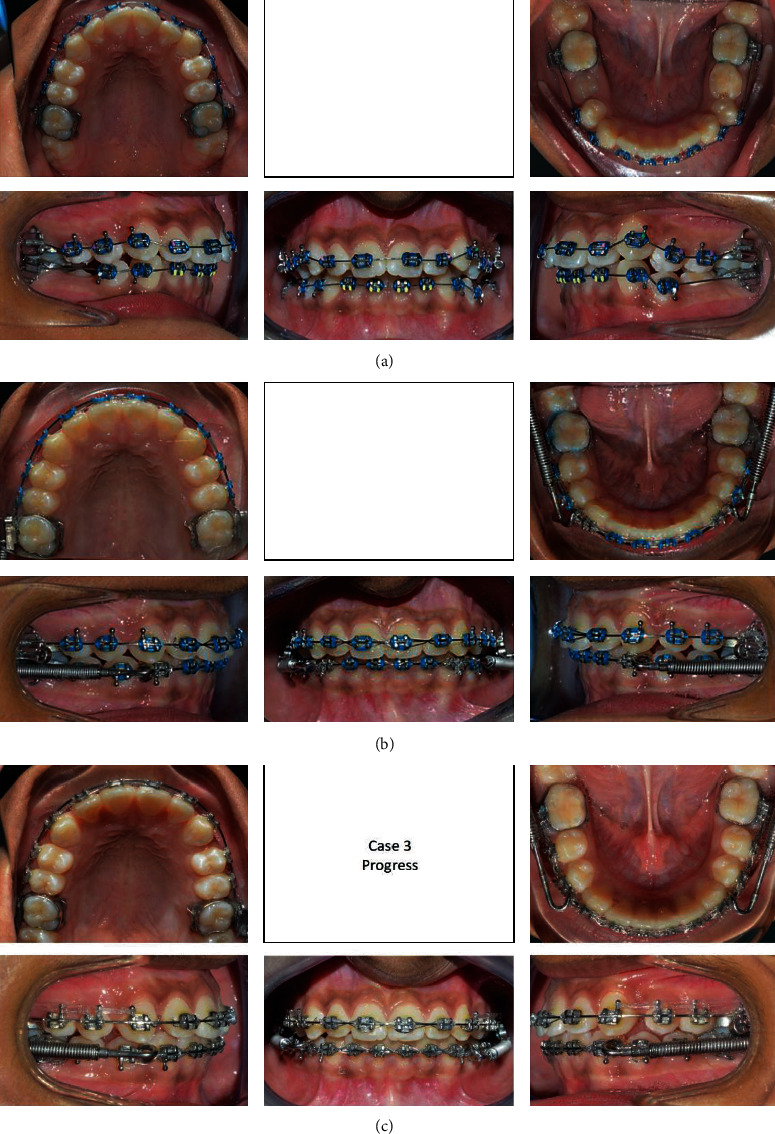
(a–c) Progress treatment intraoral photographs of the patient.

**Figure 17:**
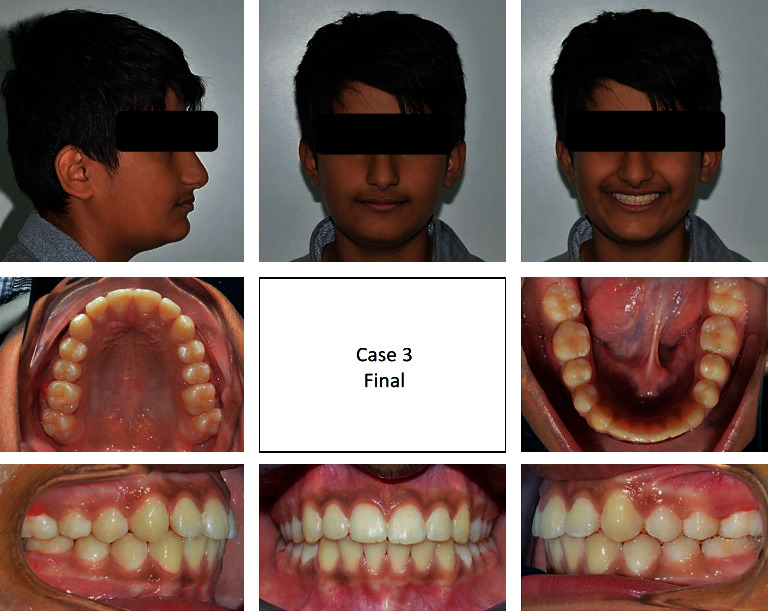
Posttreatment extraoral and intraoral photographs of the patient.

**Figure 18:**
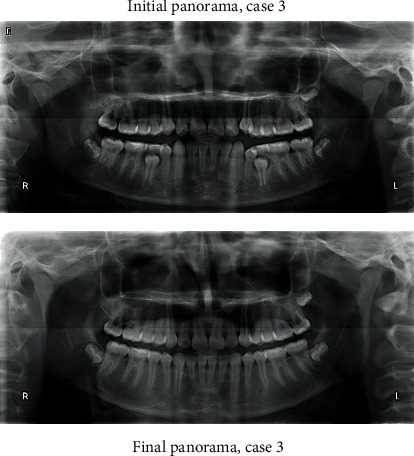
Initial and final panoramic radiographs.

**Figure 19:**
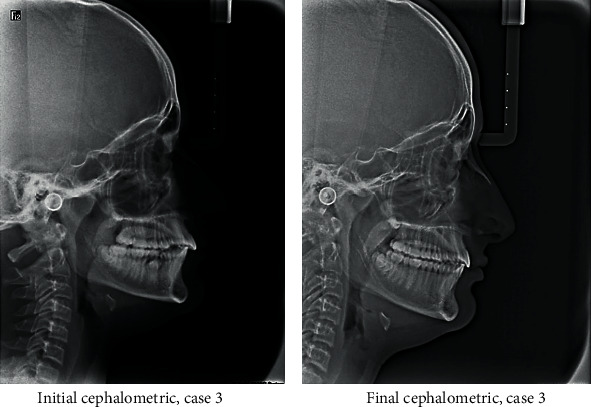
Initial and final cephalometric radiographs.

**Figure 20:**
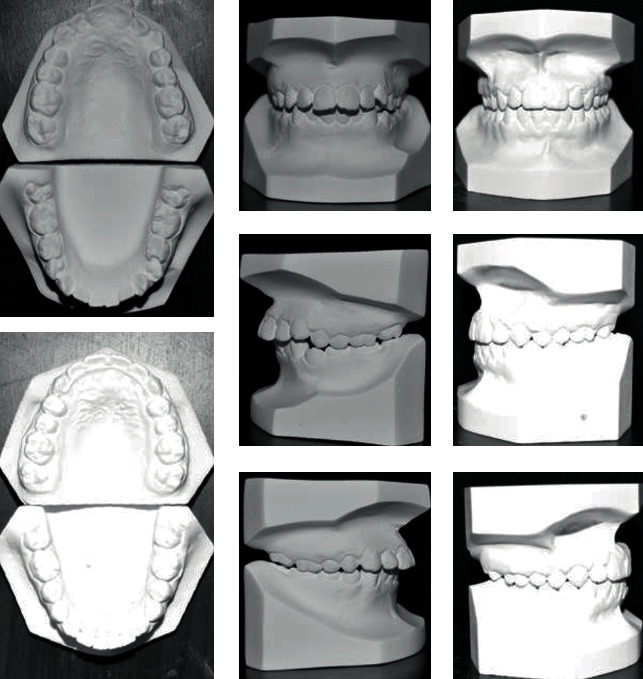
Initial and final study cast.

**Figure 21:**
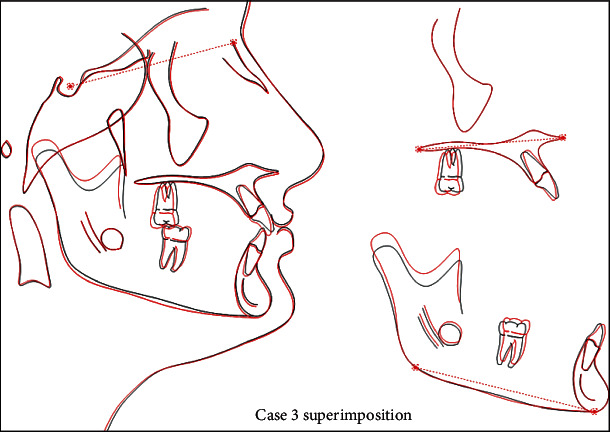
Superimposition.

A 13-year-old male patient was presented with a class II division 1 incisor relationship on mild class II skeletal base and decreased vertical proportions. This was complicated with mild crowding in the upper and lower arches and 6 mm over jet. Molar and canine relations were class II on both sides. Because the patient was still growing, treatment plan was aimed at achieving skeletal and dental correction using nonextraction protocol. Functional appliances were the method to achieve these corrections. Forsus fixed functional appliance was chosen as it requires minimal patient cooperation and can increase the lower anterior facial height and achieve proper proclination of the retruded lower incisors. Positive response for treatment was found, and both skeletal and dental problems were corrected by in increasing the lower anterior facial height, reducing overjet, and correction of class II molar and canine relation (Figures 15–21).

## 3. Conclusion

Molar distalization is one of the fundamental approaches for nonextraction therapy, especially in class II malocclusion cases. Many methods have been utilized to achieve molar distalization, including extraoral app and intraoral appliances, either removable or fixed. Innovative devices continue to evolve as the trend changing from headgears to intraoral appliances that attempt to favorably distalize teeth without requiring much patient compliance. To ensure good results, a comprehensive clinical assessment of the case must be done to understand which method will mostly be suitable for this case in terms of cost, cooperation, treatment time, and stability of the results.
